# Circular RNA Circ_0079226 Plays an Oncogenic Role in Gastric Cancer via the miR-155-5p/FOXK1/AKT Pathway

**DOI:** 10.1155/ancp/6619550

**Published:** 2025-02-13

**Authors:** Hui Zhang, Zhisheng Huang, Yingyun Zhong

**Affiliations:** Department of Gastroenterology, Panyu Maternal and Child Care Service Centre of Guangzhou (Panyu Hexian Memorial Hospital of Guangzhou), Guangzhou, Guangdong, China

**Keywords:** circ_0079226, FOXK1, gastric cancer, metastasis, miR-155-5p, tumor growth

## Abstract

**Background:** Circular RNA (circRNA) is implicated in various biological processes, including the progression of gastric cancer (GC). The specific functions and underlying mechanisms of circ_0079226 in GC are unknown.

**Methods:** We examined cancerous and adjacent noncancerous tissues from 25 patients with GC to evaluate circ_0079226, miR-155-5p, and forkhead transcription factor K1 (FOXK1) expression. Pearson's correlation analysis was used to assess the relationships among these RNAs. We examined their functional roles utilizing in vitro (cell cytotoxicity kit-8, wound healing, and Transwell invasion assays) and in vivo (xenograft mouse models) approaches. Molecular mechanisms were investigated using bioinformatics, dual-luciferase reporter assays, and rescue experiments, while quantitative real-time PCR, western blot, immunohistochemistry (IHC), fluorescence in situ hybridization (FISH), and protein immunofluorescence (IF) were used to detect gene expression.

**Results:** We found that circ_0079226 and FOXK1 levels were elevated, while miR-155-5p was reduced in GC tissues and cells. An inverse correlation existed between FOXK1 and miR-155-5p, while a direct correlation was observed between FOXK1 and circ_0079226. Circ_0079226 facilitated GC cell proliferation, migration, invasion, and in vivo tumor growth. It functions by sequestering miR-155-5p, which directly targets FOXK1. High miR-155-5p expression mitigated the effects of circ_0079226 on GC cells, and the reintroduction of FOXK1 reversed the inhibitory effects of miR-155-5p. Circ_0079226 boosts FOXK1 and its associated downstream signaling pathways, including FAK, AKT, and p-AKT, through competitive binding with miR-155-5p.

**Conclusions:** In conclusion, circ_0079226 is implicated in GC cell proliferation and metastasis by modulating the miR-155-5p/FOXK1/AKT pathway, presenting it as a potential therapeutic target.

## 1. Introduction

Gastric cancer (GC) is a significant global public health challenge, marked by a bleak 5-year survival rate of <30% [1]. Symptoms of GC usually manifest only in advanced stages, complicating early diagnosis [2]. Despite advancements in clinical diagnostics and treatment methods, the prognosis for patients with advanced GC is poor [3]. Therefore, there is an urgent need for reliable early diagnostic biomarkers and effective treatment strategies for GC.

Circular RNAs (circRNAs) constitute a unique class of noncoding RNA molecules characterized by their circular structure arising from the formation of a covalent bond. This structure lacks a 5′ end cap and a 3′ end poly (A) tail [4]. These circRNAs originate from a back-splicing of precursor mRNA (pre-mRNA) in various eukaryotic organisms. Their resistance to RNA exonuclease enhances their stability and reduces susceptibility to degradation [5]. Recent research has increasingly emphasized the significant role of circRNAs in GC progression. Wu et al. [6] reported that circRNA_0005075 may inhibit GC tumorigenesis by suppressing cell growth and metastasis. However, Miao et al. [7] provided evidence that hsa_circ_0136666 may promote GC tumor proliferation and aid in establishing a tumor microenvironment conducive to immune evasion. A specific circRNA, circ_0079226 (1221 bp), originates from the forkhead transcription factor K1 (FOXK1) gene located on chromosome 7 (positions 4721929–4795175). Previous studies reported that FOXK1 is associated with the onset of GC [8, 9]. The underlying mechanisms through which circ_0079226 contributes to GC progression are unclear and warrant further investigation.

The competing endogenous RNA (ceRNA) hypothesis posits a pivotal role for circRNAs in cancer by acting as molecular sponges for microRNAs (miRNAs), thus, affecting mRNA levels [10]. The circBase database predicts that circ_0079226 has a sequence binding site for miR-155-5p, with the highest comprehensive score (https://circinteractome.irp.nia.nih.gov/api/v2/mirnasearch?circular_rna_query=hsa_circ_0079226&mirna_query=hsa-miR-155&submit=miRNA+Target+Search). Emerging research reveals that miR-155-5p is essential in controlling cell proliferation, motility, and invasiveness across several human cancers, including colon [11], cervical [12], and clear cell renal cell carcinoma [13]. Additionally, miR-155-5p inhibits disease progression by suppressing cell growth and inducing apoptosis [14]. The long noncoding RNA AFAP1-AS1 is known for its direct interaction with miR-155-5p, which enhances cell proliferation, migration, and invasion in GC [15]. Furthermore, FOXK1, a FOX transcription factor family member, is implicated in promoting GC cell malignancy by regulating autophagy through the PI3K/AKT/mTOR pathway [16]. Zhang et al. [9] reported that co-expression of FOXK1 with vimentin enhances metastatic potential by facilitating epithelial–mesenchymal transition (EMT) in GC cells. However, the exact interaction between miR-155-5p and FOXK1 within the circ_0079226 regulatory network is unknown. CircRNA interactome and starBase predictions indicate that circ_0079226 and FOXK1 share miR-155-5p binding sites. Therefore, we propose a potential ceRNA network comprising circ_0079226, miR-155-5p, and FOXK1, which may clarify the role of circ_0079226 in GC.

This study examined circ_0079226, miR-155-5p, and FOXK1 expression in GC tissues and analyzed their interrelationships. We evaluated the effects of circ_0079226 on cell growth, movement, invasiveness, and tumor formation in GC models through a comprehensive set of functional assays. Furthermore, this study aimed to investigate the potential mechanisms facilitated by circ_0079226 involving miR-155-5p and FOXK1.

## 2. Materials and Methods

### 2.1. Tissue Samples

Tissue samples were obtained from 25 pairs of cancerous and adjacent noncancerous tissues (normal tissue adjacent to the tumor) from patients diagnosed with GC after radical gastrectomy at the Affiliated Hexian Memorial Hospital of Southern Medical University. Not all patients received chemotherapy or radiotherapy treatment before surgery. One section was rapidly frozen in liquid nitrogen and stored at −80°C, while others were fixed in formalin, embedded in paraffin, and prepared for immunohistochemistry (IHC) studies. The patients provided written informed consent before participating in the study. This study was approved by the ethics committee of Panyu Maternal and Child Care Service Centre of Guangzhou (Panyu Hexian Memorial Hospital of Guangzhou) with approval number No. 2022071408, and all procedures involving human participants were conducted in accordance with the Declaration of Helsinki and the ethical standards of the institutional and national research committee.

### 2.2. Cell Culture

The human GC cell lines MKN74, NCI-N87, KATO III, MKN-7, HGC-27, and the normal gastric epithelial cell line GES-1 were obtained from the American Type Culture Collection (Manassas, VA, USA). Cell lines were validated by the Genomics Unit at ClMA through short tandem repeat profiling utilizing the AmpFLSTR ldentifler Plus PCR Amplification Kit. The cells were cultured in RPMI-1640 medium (Servicebio, Wuhan, China) supplemented with 10% fetal bovine serum (Thermo Fisher Scientific, Waltham, MA, USA) and 1% penicillin–streptomycin (Servicebio), maintained at 37°C in a 5% CO_2_ environment.

### 2.3. Fluorescence In Situ Hybridization (FISH)

The procedure for preparing the cell slides involved fixation with 4% paraformaldehyde for 20 min, followed by the administration of proteinase K (Cat: P1120, Solarbio, Beijing, China) at 20 μg/mL for enzymatic digestion. Subsequently, hybridization was performed using the hsa-circ_0079226 probe (Sangon Biotech, Shanghai, China) at 8 ng/μL, and this process was permitted to continue overnight. Subsequent to hybridization, the slides were incubated with an anti-digoxigenin-horseradish peroxidase (HRP) antibody (Boster, Wuhan, China). For nuclear visualization, 4′, 6-diamidino-2-phenylindole (DAPI, Solarbio) was used for counterstaining. The concluding step involved acquiring images using an immunofluorescence (IF) microscope (Leica, TCS SP5II).

### 2.4. IF Assay

Cells were cultured on glass slides in petri dishes until they achieved 80% confluence. Cells were initially fixed with 4% paraformaldehyde for 10 min, followed by permeabilization with 0.5% Triton X-100 for 15 min. After blocking with 3% BSA for 30 min at 27°C, the specimens were incubated overnight at 4°C with a primary FOXK1 antibody (ab18196, 1:500, Abcam, Cambridge, MA, USA). A FITC-conjugated secondary antibody (Boster) was administered for 45 min at 37°C. DAPI was utilized for nuclear staining before imaging with a Leica TCS SP5II IF microscope.

### 2.5. Cellular Transfection

Short hairpin RNA (shRNA) vectors targeting circ_0079226 (sh-circ_0079226) and a control shRNA (shCtrl), miR-155-5p mimic and its negative control (NC), and overexpression vectors for circ_0079226 and FOXK1 and a control vector were obtained from GenePharma Co., Ltd. (Shanghai, China). The transfection of GC cells was performed using Lipofectamine 3000 (Thermo Fisher Scientific) in accordance with the manufacturer's instructions.

### 2.6. Dual-Luciferase Reporter Assay

Wild-type (WT) and mutant-type (MUT) sequences of circ_0079226 and the 3′UTR of FOXK1, corresponding to their interaction sites with miR-155-5p, were inserted into the pGL3 luciferase reporter vector (Promega, Madison, WI, USA), creating circ_0079226-WT/MUT and FOXK1 3′UTR-WT/MUT vectors. HEK293T cells were subsequently cotransfected with these vectors and either the miR-155-5p mimic or its NC for 48 h. Luciferase activity was measured using the dual-luciferase reporter assay system (Promega), with firefly luciferase activity standardized against renilla luciferase activity to ensure precise results.

### 2.7. Cell Counting Kit-8 (CCK-8) Assay

Cell viability was evaluated using a CCK-8 assay kit (#CK04, Dojindo, Tokyo, Japan) according to the manufacturer's instructions. Transfected cells were seeded in 96-well plates and incubated in a fresh medium for 24 h. Subsequently, 10 µL of CCK-8 solution was introduced to each well and incubated for 2 h at 37°C. The absorbance was measured at 450 nm.

### 2.8. Wound Healing Assay

Cell migration was evaluated using a wound healing assay. After transfection, cells were seeded into six-well plates, and two parallel scratches were created using a sterile 200 µL pipette tip. Subsequent to two washes with phosphate-buffered saline, the cells were incubated in a serum-free medium for 24 h. Microscopic images of the scratches were obtained initially and after 24 h at 100x magnification. Migration was calculated using the formula: (initial wound width − wound width at 24 h)/initial wound width, expressed as a percentage.

### 2.9. Transwell Invasion Assay

We used a Matrigel-coated Transwell Boyden Chamber (Corning Incorporated, Corning, NY, USA) with 8 μm pores to evaluate invasiveness. Cells in serum-free medium were introduced into the upper chamber, while medium containing 10% fetal bovine serum was added to the lower chamber. After 24 h, noninvasive cells were eliminated from the upper membrane. The cells on the lower membrane were fixed with methanol, stained with crystal violet, and subsequently imaged for quantification using an inverted Olympus microscope (Olympus, Tokyo, Japan).

### 2.10. Xenograft Tumor Models

We obtained 20 male BALB/c athymic nude mice (6–8 weeks; 22–25 g) from Shanghai Charles River Co. (Shanghai, China). The mice were housed in sterile, individually ventilated cages under a 12 h light/dark cycle, with ad libitum water and food. These mice received subcutaneous injections on both flanks with 2 × 10^6^ NCI-N87 and KATO III cells to create xenograft models, transfected with sh-circ_0079226 and shCtrl lentiviruses and mixed with Matrigel (*n* = 5 per group). Tumor sizes were recorded every 3 days starting from day 10. On day 31, tumors were excised for analysis using quantitative real-time PCR (qRT-PCR), western blot, and IHC assays. The ethics board of Guangzhou Yongnuo Biomedical Experimental Animal Center approved this procedure (No. IACUC-AEWC-F240621077).

### 2.11. qRT-PCR

Total RNA was extracted from tissues and cells using Trizol reagent (Thermo Fisher Scientific). Mature miR-155-5p expression was measured using TaqMan MicroRNA Assays (Thermo Fisher Scientific), with U6 as the internal control. For circ_0079226 and FOXK1 quantification, reverse transcription with M-MLV reverse transcriptase was followed by PCR using SYBR Green Master Mix (Thermo Fisher Scientific). The PCR involved an initial 30 s pre-denaturation at 95°C, followed by 40 cycles of 5 s denaturation at 95°C and 30 s annealing at 60°C. GAPDH served as the normalization reference gene for circ_0079226 and FOXK1 expression.  Table 1 presents the primer sequences.

### 2.12. Western Blot Analysis

Protein extraction utilized RIPA lysis buffer (Beyotime, Shanghai, China) supplemented with protease inhibitors. Protein concentrations were measured using the BCA kit (Beyotime). Subsequently, 30 μg of protein was subjected to 10% SDS-PAGE and subsequently transferred to PVDF membranes (Millipore, Billerica, CA, USA). Membranes were blocked using 5% nonfat dried milk for 2 h, followed by overnight incubation at 4°C with primary antibodies targeting FOXK1 (ab18196, 1:2000, Abcam), FAK (ab40479, 1:2000, Abcam), AKT (ab8805, 1:2000, Abcam), p-AKT (ab38449, 1:1000, Abcam), E-cadherin (ab 40772, 1:5000, Abcam), N-cadherin (ab76011, 1:5000, Abcam), vimentin (ab8978, 1:5000, Abcam), and GAPDH (ab8245, 1:5000, Abcam). HRP-conjugated secondary antibodies (Boster) were utilized for 2 h at room temperature. Proteins were detected using an enhanced chemiluminescence kit (Beyotime), and ImageJ software was used to quantify the protein expression levels.

### 2.13. IHC Assay

IHC assay was used to assess the FOXK1 and Ki-67 expression in human and mouse tumor tissues. Tissues were fixed in 4% paraformaldehyde, embedded in paraffin, and sectioned into 4 µm sections. Antigen retrieval was performed in citrate buffer at 95°C for 20 min. Slides were treated with 3% hydrogen peroxide to block endogenous peroxidase, followed by treatment with 5% goat serum for blocking. Primary antibodies for FOXK1 (ab18196, 1:500, Abcam) and Ki-67 (ab15580, 1:500, Abcam) were incubated overnight at 4°C. Sections were subsequently washed, treated with biotinylated secondary antibodies and peroxidase-conjugated streptavidin (Boster), and developed with DAB for 5 min, followed by hematoxylin counterstain. The slides were examined under a light microscope.

### 2.14. Statistical Analysis

Experiments were performed at least thrice. Data are represented as mean ± standard deviations from three or more biological samples. GraphPad Prism software (version 8.0) was used for scatter plots and histogram analyses. Student's *t*-test and one-way analysis of variance were used to determine statistical significance. Pearson's correlation was used for RNA level relationships, setting significance at *p*  < 0.05.

## 3. Results

### 3.1. Evaluating Expression Patterns and Associations Among circ_0079226, miR-155-5p, and FOXK1 in GC Specimens

**Figure 1:**
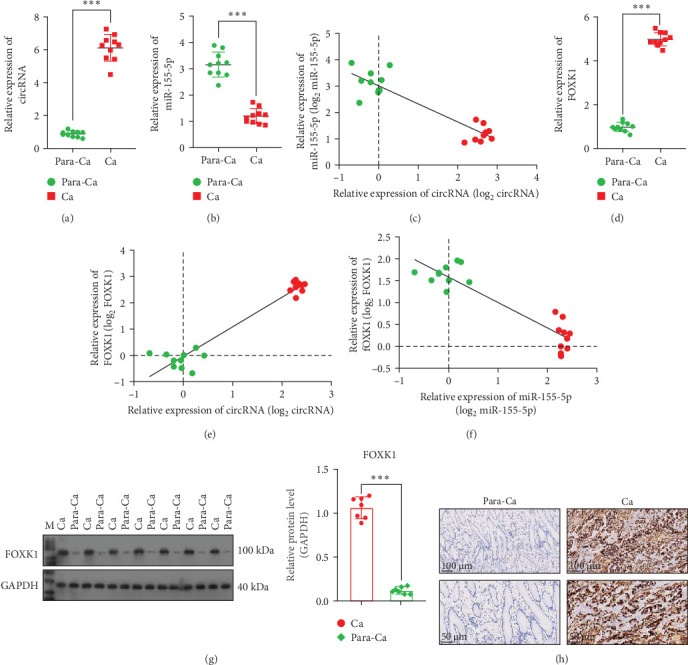
Evaluating expression patterns and associations among circ_0079226, miR-155-5p, and forkhead transcription factor K1 (FOXK1) in gastric cancer (GC) specimens. (A, B) Expression levels of circ_0079226 and miR-155-5p were determined in 10 pairs of cancerous and para-cancerous tissues from patients with GC using quantitative real-time PCR (qRT-PCR) analysis. (C) Pearson's correlation analysis was used to analyze the linear correlation between circ_0079226 and miR-155-5p in cancer tissues (*n* = 10). (D) The mRNA levels of FOXK1 were measured in cancerous and para-cancerous tissues (*n* = 10), whose correlation with circ_0079226 (E) and miR-155-5p (F) was evaluated using Pearson's correlation analysis. (G) FOXK1 protein level was tested using western blot in cancerous and para-cancerous tissues (*n* = 7). *⁣*^*∗∗∗*^*p*  < 0.001, compared with para-cancerous tissues. (H) Immunohistochemistry (IHC) measured in situ FOXK1 expression, and the scale bar was 100 μm (upper) and 50 μm (below) in IHC images.

Table 2 presents the clinical and pathological characteristics of patients. The qRT-PCR analysis revealed a significant increase in circ_0079226 expression in cancerous tissues of patients with GC compared to adjacent noncancerous tissues (Figure 1A). However, miR-155-5p levels were significantly lower in GC tissues than in para-cancerous tissues (Figure 1B). The reduction in miR-155-5p was inversely proportional to circ_0079226 expression within the cancerous tissues (Figure 1C). Furthermore, the mRNA expression of FOXK1 was assessed in these tissue samples. Consistent with circ_0079226 expression patterns, FOXK1 mRNA was significantly upregulated in GC tissues (Figure 1D). The increase in expression was positively correlated with circ_0079226 levels (Figure 1E) and negatively correlated with miR-155-5p levels in the cancerous tissues (Figure 1F). The increased expression of FOXK1 was further corroborated by western blot analysis (Figure 1G) and IHC assay (Figure 1H), which confirmed its upregulation in GC tissues compared to para-cancerous tissues.

### 3.2. Investigating the Expression Profiles and Targeting Relationships of circ_0079226, miR-155-5p, and FOXK1 in GC Cells

**Figure 2:**
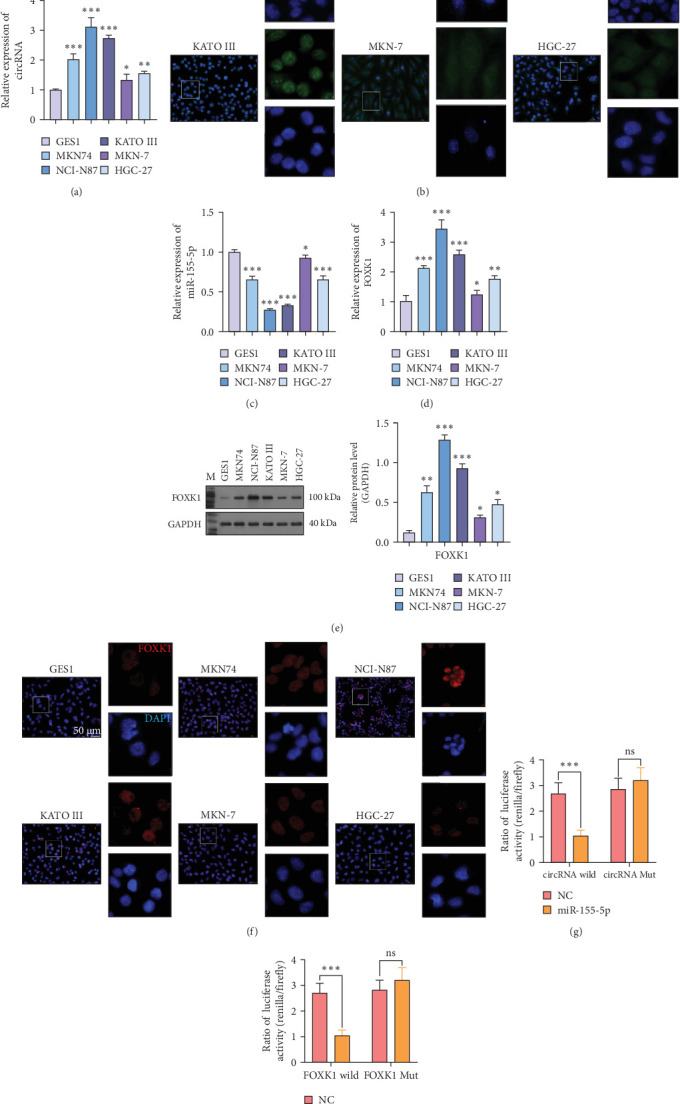
Investigating the expression profiles and targeting relationships of circ_0079226, miR-155-5p, and forkhead transcription factor K1 (FOXK1) in gastric cancer (GC) cells. The quantitative real-time PCR (qRT-PCR) analysis (A) and fluorescence in situ hybridization (FISH) assay (B) were performed to determine circ_0079226 expression levels in five GC cell lines (MKN74, NCI-N87, KATO III, MKN-7, and HGC-27) and the normal human gastric epithelial cell line GES-1. (C, D) The qRT-PCR analysis was performed to analyze miR-155-5p and FOXK1 expression levels in the above cell lines. (E, F) FOXK1 protein levels were detected using western blot analysis and immunofluorescence (IF) assay in the above cell lines. *⁣*^*∗*^*p*  < 0.05, *⁣*^*∗∗*^*p*  < 0.01, *⁣*^*∗∗∗*^*p*  < 0.001, compared with GES-1. A dual-luciferase reporter assay was used to confirm the interaction between miR-155-5p and circ_0079226 (G) and between miR-432-5p and the 3′UTR of FOXK1 (H). *⁣*^*∗∗∗*^*p*  < 0.001. Three repetitions were performed in each experiment, with three parallels every time.

Consistent with tissue sample observations, circ_0079226 exhibited a significant upregulation in all five analyzed GC cell lines (MKN74, NCI-N87, KATO III, MKN-7, and HGC-27) relative to the normal GES-1 cell line. The upregulation was validated using qRT-PCR (Figure 2A) and FISH assays (Figure 2B). A decrease in miR-155-5p expression was observed in these cell lines (Figure 2C). FOXK1 expression was significantly reduced at mRNA and protein levels, as validated by qRT-PCR (Figure 2D), western blot (Figure 2E), and IF assay (Figure 2F). Dual-luciferase reporter assays revealed that miR-155-5p significantly reduced luciferase activity in the circ_0079226-WT group, but did not affect the circ_0079226-MUT group (Figure 2G), indicating that miR-155-5p is a target of circ_0079226. Similarly, a miR-155-5p replicate reduced luciferase activity in the FOXK1-WT group, with no significant effect on the FOXK1-MUT group (Figure 2H), indicating that FOXK1 is a downstream target of miR-155-5p. The luciferase reporter analysis confirmed that miR-155-5p interacts with the FOXK1 3′UTR (Figure 2H), indicating that circ_0079226 enhances GC cell invasion and proliferation by sequestering miR-155-5p.

### 3.3. Impact of circ_0079226 Suppression on the miR-155-5p/FOXK1 Regulatory Axis and Malignant Phenotypes in GC Cells

**Figure 3:**
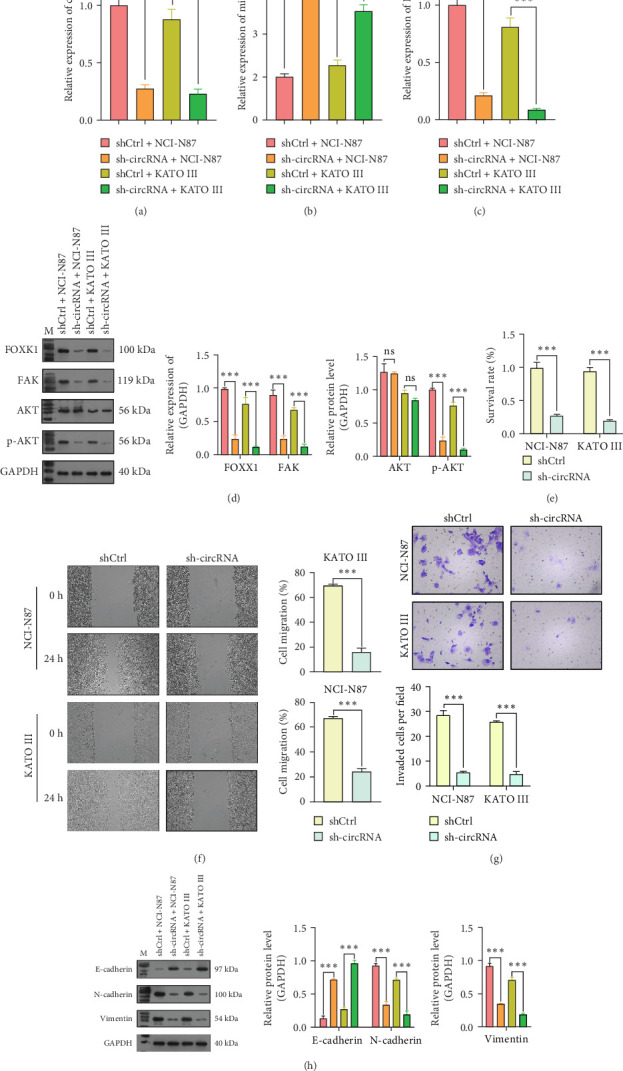
Impact of circ_0079226 suppression on the miR-155-5p/forkhead transcription factor K1 (FOXK1) regulatory axis and malignant phenotypes in gastric cancer (GC) cells. NCI-N87 and KATO III cells were transfected with sh-circ_0079226 or control short hairpin RNA (shCtrl) for 48 h. (A–C) The expression levels of circ_0079226, miR-155-5p, and FOXK1 were determined using quantitative real-time PCR (qRT-PCR) analysis in the transfected cells above. (D) FOXK1, FAK, AKT, and p-AKT protein levels were detected using western blot analysis in the transfected cells above. (E) Cell survival rate was analyzed using the cell counting kit-8 (CCK-8) assay. (F) A wound healing assay was performed to assess cell migration capacity. (G) The invasive ability was assessed using the Transwell invasion assay. (H) Expression of epithelial–mesenchymal transition (EMT)-associated protein markers (E-cadherin, N-cadherin, and vimentin) was analyzed using a western blot assay. Three repetitions were performed in each experiment, with three parallels every time. *⁣*^*∗∗∗*^*p*  < 0.001.

Because of the observed upregulation of circ_0079226 in GC cells, the NCI-N87 and KATO III cell lines, which exhibited comparatively higher expression levels of circ_0079226, were selected for loss-of-function studies. Initial qRT-PCR analysis revealed a significant reduction in circ_0079226 levels in NCI-N87 and KATO III cells transfected with sh-circ_0079226 compared to the control group (Figure 3A). Simultaneously, an increase in miR-155-5p expression was observed (Figure 3B), while the mRNA levels of FOXK1 were diminished following circ_0079226 knockdown (Figure 3C). Western blot analysis revealed that sh-circ_0079226 transfection significantly reduced FOXK1, FAK, and p-AKT expression in these cell lines (Figure 3D). Further functional assays revealed that circ_0079226 knockdown significantly decreased cell survival rates (Figure 3E) and the numbers of migratory (Figure 3F) and invasive cells (Figure 3G) in NCI-N87 and KATO III cells. This knockdown led to increased E-cadherin expression and decreased N-cadherin and vimentin levels (Figure 3H), highlighting the effects of circ_0079226 on the malignant cellular behavior and molecular dynamics in GC cells.

### 3.4. Knockdown of circ_0079226 Inhibited GC Tumor Growth In Vivo

**Figure 4:**
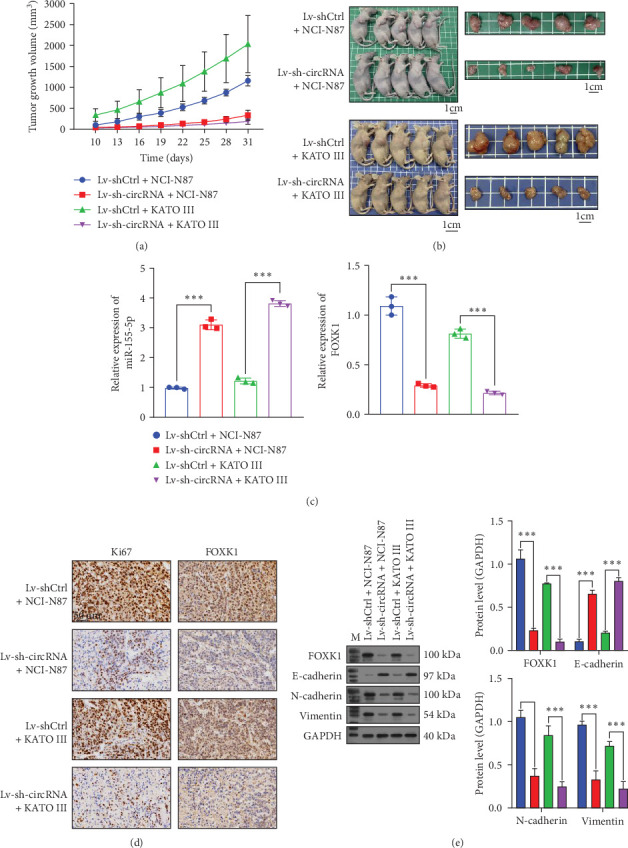
Knockdown of circ_0079226 inhibited gastric cancer (GC) tumor growth in vivo. (A) Volume of xenografted tumors derived from NCI-N87 and KATO III cells transfected with lentiviruses carrying sh-circ_0079226 or control short hairpin RNA (shCtrl; *n* = 5 each group). (B) Representative images of subcutaneously injecting nude mice and xenograft tumors. (C) Quantitative real-time PCR (qRT-PCR) analysis of miR-155-5p and forkhead transcription factor K1 (FOXK1) in xenografted tumors derived from NCI-N87 and KATO III cells. (D) Immunohistochemistry (IHC) assays for xenografted tumors derived from NCI-N87 and KATO III cells stained with Ki-67 antibody and FOXK1 antibody, respectively. (E) FOXK1, E-cadherin, N-cadherin, and vimentin protein levels were detected using western blot analysis in xenografted tumors derived from NCI-N87 and KATO III cells. Five repetitions were performed in each experiment, with three parallels every time. *⁣*^*∗∗∗*^*p*  < 0.001.

NCI-N87 and KATO III cells, previously transfected with lentiviruses carrying sh-circ_0079226 or shCtrl, were implanted into nude mice to investigate the in vivo role of circ_0079226 in GC. The tumors in the sh-circ_0079226 group exhibited a significant decrease in volume (Figure 4A) and size (Figure 4B) than those in the shCtrl group. qRT-PCR analysis revealed an upregulation of miR-155-5p and a downregulation of FOXK1 in the sh-circ_0079226 group compared with the shCtrl group (Figure 4C). IHC assay revealed a significant decrease in FOXK1 and Ki-67 protein levels in the sh-circ_0079226 group within the subcutaneous tumors (Figure 4D). Corresponding with the downregulation of circ_0079226, an increase in E-cadherin protein level and a reduction in FOXK1, N-cadherin, and vimentin levels were observed in the tumor tissues (Figure 4E). These findings revealed that circ_0079226 knockdown impedes GC tumor growth in vivo.

### 3.5. Enhanced miR-155-5p Expression Mitigated the Impact of circ_0079226 on the Proliferation, Migration, and Invasion of GC Cells

**Figure 5:**
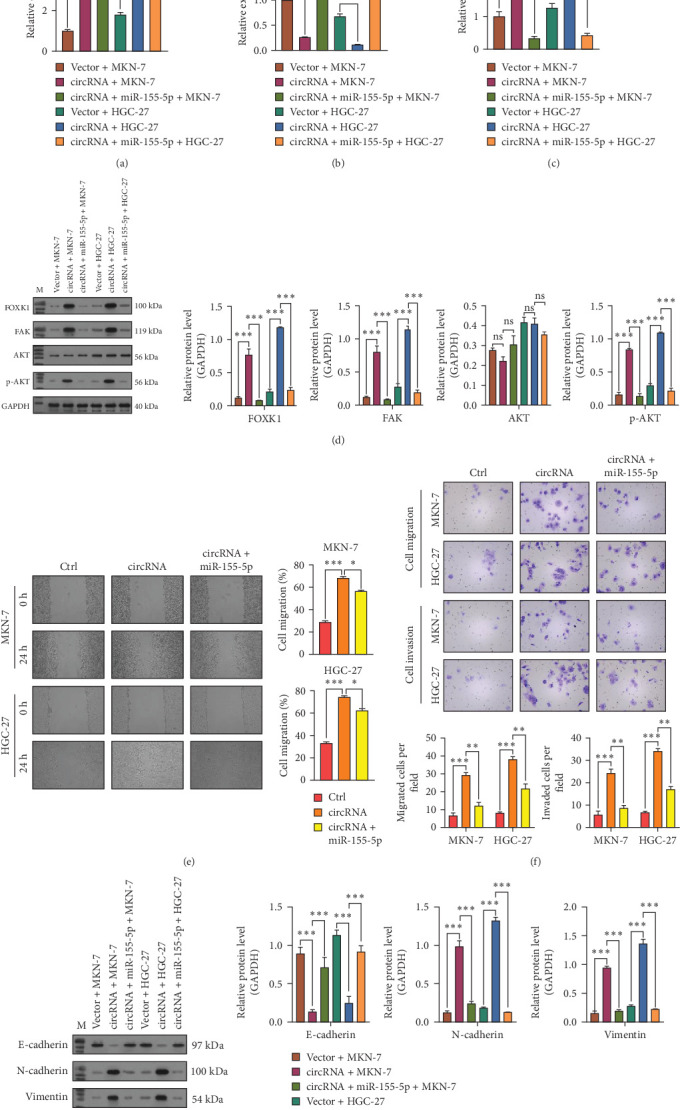
Enhanced miR-155-5p expression mitigated the impact of circ_0079226 on the proliferation, migration, and invasion of gastric cancer (GC) cells. MKN-7 and HGC-27 cells were transfected with a control vector, circ_0079226 alone, or circ_0079226 combined with a miR-155-5p mimic for 48 h. (A–C) Forkhead transcription factor K1 (FOXK1), circ_0079226, and miR-155-5p expression levels were determined using quantitative real-time PCR (qRT-PCR) analysis in the transfected cells above. (D) FOXK1, FAK, AKT, and p-AKT protein levels were detected using western blot analysis in the transfected cells above. (E) A wound healing assay was implemented to assess cell migration capacity. (F) The invasive ability was assessed using the Transwell invasion assay. (G) The expression of epithelial–mesenchymal transition (EMT)-associated protein markers (E-cadherin, N-cadherin, and vimentin) was analyzed using a western blot assay. Three repetitions were performed in each experiment, with three parallels every time. *⁣*^*∗*^*p*  < 0.05, *⁣*^*∗∗*^*p*  < 0.01, *⁣*^*∗∗∗*^*p*  < 0.001.

MKN-7 and HGC-27 cells, which exhibit relatively lower expressions of circ_0079226, were transfected with a control vector, circ_0079226 alone, or circ_0079226 combined with a miR-155-5p mimic to assess the necessity of miR-155-5p for the circ_0079226-mediated regulatory mechanism. qRT-PCR analysis revealed that overexpressing miR-155-5p did not change the increased circ_0079226 levels in MKN-7 and HGC-27 cells (Figure 5A). However, it effectively neutralized the suppressive effect of circ_0079226 on miR-155-5p expression (Figure 5B) and reversed the upsurge in FOXK1 mRNA caused by circ_0079226 (Figure 5C). Additionally, miR-155-5p overexpression decreased the circ_0079226-induced increase in FOXK1 protein and its downstream targets, including FAK and p-AKT (Figure 5D). Functional assays revealed that miR-155-5p overexpression reinstated the migration (Figure 5E) and invasion (Figure 5F) capacities in MKN-7 and HGC-27 cells, which were increased by circ_0079226. Moreover, the effect of circ_0079226 on reducing E-cadherin protein levels and increasing N-cadherin and vimentin levels was mitigated by miR-155-5p overexpression (Figure 5G).

### 3.6. FOXK1 Overexpression Reversed miR-155-5p-Induced Cell Growth and Migration Inhibition In Vitro

**Figure 6:**
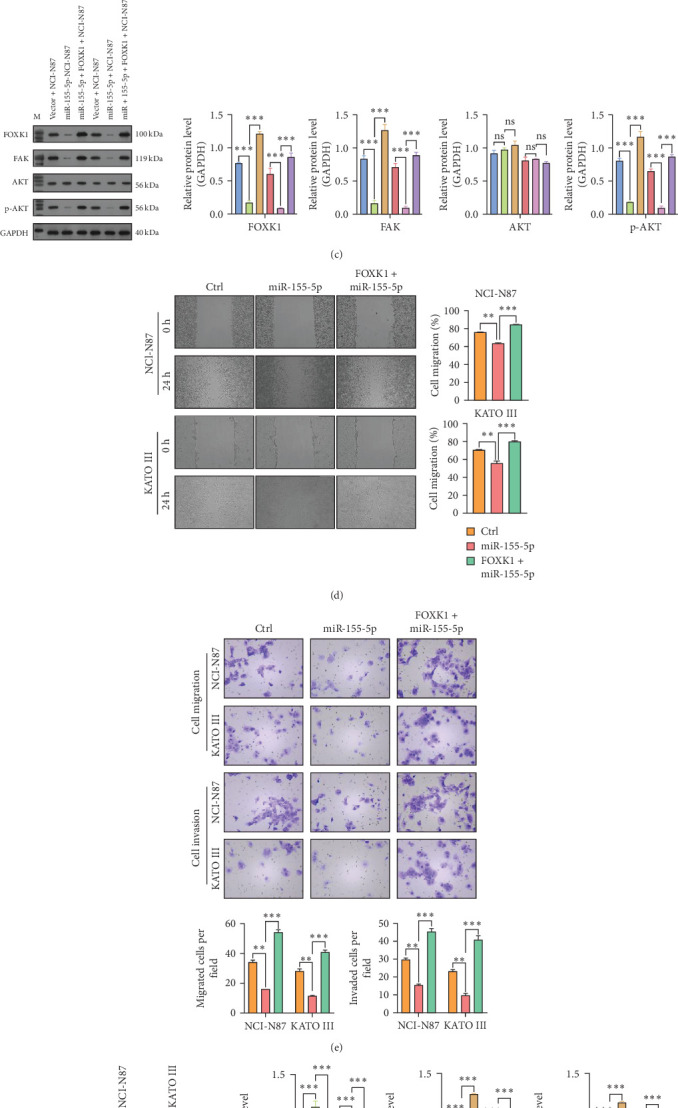
Restoration of forkhead transcription factor K1 (FOXK1) diminished the suppressive impact of miR-155-5p on the proliferation, migration, and invasion of gastric cancer (GC) cells. NCI-N87 and KATO III cells were cotransfected with miR-155-5p mimic and FOXK1 overexpression plasmid for 48 h. (A, B) FOXK1 and miR-155-5p expression levels were determined using quantitative real-time PCR (qRT-PCR) analysis in the transfected cells above. (C) FOXK1, FAK, AKT, and p-AKT protein levels were detected using western blot analysis in the transfected cells above. (D) A wound healing assay was implemented to assess cell migration capacity. (E) The invasive ability was assessed using the Transwell invasion assay. (F) The expression of epithelial–mesenchymal transition (EMT)-associated protein markers (E-cadherin, N-cadherin, and vimentin) was analyzed using western blot assay. Three repetitions were performed in each experiment, with three parallels every time. *⁣*^*∗∗*^*p*  < 0.01, *⁣*^*∗∗∗*^*p*  < 0.001.

We performed rescue experiments in NCI-N87 and KATO III cells by cotransfecting them with a miR-155-5p replicate and a FOXK1 overexpression plasmid to investigate if FOXK1 is a downstream target in miR-155-5p-mediated GC cell function regulation. qRT-PCR revealed that FOXK1 overexpression did not alter miR-155-5p upregulation in either cell line (Figure 6A). However, it significantly counteracted the inhibitory effect of miR-155-5p on FOXK1 expression (Figure 6B), indicating that FOXK1 is a downstream target of miR-155-5p. Additionally, the increased FOXK1 levels counteracted FOXK1 protein reduction and its downstream targets, including FAK, AKT, and phosphorylated AKT, which were initially decreased by miR-155-5p overexpression (Figure 6C). Wound healing assay and Transwell invasion assay illustrated that FOXK1 overexpression restored the proliferation and migration abilities of NCI-N87 and KATO III cells after miR-155-5p upregulation (Figure 6D,E). Besides, western blot analysis revealed that FOXK1 upregulation recovered the EMT in NCI-N87 and KATO III cells after miR-155-5p overexpression (Figure 6F).

## 4. Discussion

circRNAs are distinguished by their covalently closed loop structure, which enhances their stability relative to linear RNAs [4]. Previous studies reported the significant roles of circRNAs in various biological functions, particularly in cancer development and metastasis [17–19]. However, the specific functions and regulatory mechanisms of circRNAs in GC are unclear. This study focuses on circ_0079226, derived from the FOXK1 gene on chromosome 7 (positions 4721929–4795175) and demonstrated a significant increase in its expression in GC tissues compared to adjacent noncancerous tissues. This finding is consistent with those of previous studies that reported that circ_0079226 exhibits enhanced stability over its linear RNA counterpart, indicating resistance to RNase R degradation. In vivo, functional assays revealed that circ_0079226 facilitates cell growth, motility, and invasiveness in GC. Furthermore, in vivo experiments using nude mice further confirmed the role of circ_0079226 in promoting tumor growth.

Additionally, circRNAs are predominantly known for their role as “miRNA sponges,” which bind to miRNAs and inhibit their activity, thereby, preventing the miRNA-mediated degradation of target genes [20]. This study confirmed the role of circ_0079226 as a “miRNA sponge” through its interaction with miR-155-5p in GC cells. Statistical analysis revealed an inverse correlation between circ_0079226 and miR-155-5p expression levels in GC tissues. Additionally, rescue experiments demonstrated that increasing miR-155-5p expression could mitigate the effects of circ_0079226 on GC cell proliferation, migration, and invasion, highlighting the role of circ_0079226 in impeding GC progression by absorbing the tumor-suppressive miR-155-5p. This phenomenon is observed in other cancers, including cervical cancer with circ_0000337 [21] and esophageal squamous cell carcinoma (ESCC) with circ_0000592 [22], both acting similarly to affect disease progression. These findings are consistent with those of earlier research, which reported a decrease in miR-155-5p in GC associated with advanced tumor stages and metastasis [23]. Zhu et al. [24] revealed that miR-155-5p downregulation causes BM-MSCs to resemble GC-MSCs and identified the oncogenic function of hsa_circ_0001829 in GC, both functioning as miR-155-5p sponges. In addition, Yu et al. [25] reported that in liver cancer, miR-155-5p targets and regulates ATG5, thereby, enhancing the sensitivity of liver cancer cells to adriamycin through its effects on autophagy. In Wilms tumor, miR-155-5p demonstrated an antitumor effect by targeting IGF2 [26]. However, miR-155-5p was involved in the interaction between cancer cells and tumor-associated fibroblasts in colorectal cancer, contributing to the promotion of cancer cell metastasis [27]. The diversity of miRNA target genes may be the primary reason influencing the different roles of miR-155-5p across different tumors.

Based on the knowledge that circ_0079226 functions as a sponge for miR-155-5p, we investigated the targets of miR-155-5p and identified FOXK1 as a target, which shares binding sites with circ_0079226. Our statistical analysis revealed an inverse correlation between FOXK1 and miR-155-5p and a direct correlation between FOXK1 and circ_0079226. Additionally, rescue experiments revealed that reintroducing FOXK1 could negate the inhibitory effects of miR-155-5p on cell proliferation, migration, and invasion in GC cells, highlighting the role of FOXK1 in GC progression. The FOX family of transcription factors, FOXK1, serves as a potential independent prognostic marker for patients with GC and its suppression triggers autophagy, which may reverse the EMT process in GC [28]. FOXK1 is involved in decreasing the epithelial marker E-cadherin and increasing mesenchymal markers such as vimentin, thereby, aiding tumor cell invasion in GC [29]. Furthermore, FOXK1 is a downstream target of miR-1294 and miR-593-3p, essential in promoting GC [30, 31]. The involvement of FOXK1 in activating the AKT/mTOR signaling pathway further supports its implication in the malignancy of various cancers [31, 32]. FOXK1 activates the AKT/mTOR signaling pathway and promotes hyperplasia and metastasis of gallbladder cancer [33]. Hong et al. [34] reported that FOXK1 expression is regulated by circPPP2R4/miR-646, and it promotes colorectal cancer progression. We demonstrated that the inhibition of the FOXK1/AKT signaling pathway via miR-155-5p is an essential mechanism by which circ_0079226 enhances tumor metastasis in GC. However, more patient tissue samples and follow-up data are required in future research to clarify the possibility of using circ_0079226, miR-155-5p, and FOXK1 as diagnostic or therapeutic targets in GC.

In conclusion, this study demonstrated the cancer-promoting role of circ_0079226 in GC, as evidenced by its facilitation of cellular proliferation, migration, invasion, and xenograft tumor development. These oncogenic effects are probably mediated by the modulation of miR-155-5p and FOXK1. Our results thus highlight circ_0079226 as a possible therapeutic target in GC treatment.

## Figures and Tables

**Table 1:** Primersfor qRT-PCR.

Gene	Forward (5' - 3')	Reverse (5' - 3')
circ_0079226	GCATATTGCTTCCCTTAAAACA	GCTGTCCTCGCCGACTTC
FOXK1	ATCGTAACCTCACAGCAGCC	GATGTATCCGTTGGCCGAGT
miR-155-5p	CGCGTTAATGCTAATCGTGAT	AGTGCAGGGTCCGAGGTATT
U6	CTCGCTTCGGCAGCACA	AACGCTTCACGAATTTGCGT
GAPDH	GACCACAGTCCATGCCATCA	CCGTTCAGCTCAGGGATGAC

Abbreviations: FOXK1, forkhead transcription factor K1; qRT-PCR, quantitative real-time PCR.

**Table 2:** Pathological TNM staging table of 25 gastric cancer (GC) patients.

Listings	Category	Number of people
Gender	Male	10
	Female	15

Age	>55 years old	20
	≤55 years old	5

Lymph node metastasis (positive or negative)	Positive	19
	Negative	6

TNM stage	Ⅰ	3
	Ⅱ	3
	Ⅲ	13
	Ⅳ	6

Tumor size (diameter)	>5 cm	9
	≤5 cm	16

## Data Availability

The data that support the findings of this study are available from the corresponding author upon reasonable request.

## Nomenclature

circRNA: circular RNA
GC: gastric cancer
FOXK1: forkhead transcription factor K1
ceRNA: competing endogenous RNA
EMT: epithelial–mesenchymal transition
IHC: immunohistochemistry
qRT-PCR: quantitative real-time PCR.
